# Probiotic mediated modulation of neonatal health: a meta-analysis of prematurity-related morbidity, jaundice, and respiratory distress

**DOI:** 10.3389/fcimb.2026.1866590

**Published:** 2026-08-19

**Authors:** Lifang Lu, Juan Li, Xiajuan Yao

**Affiliations:** 1 Department of Neonatology, Suzhou Ninth People’s Hospital, Suzhou, Jiangsu, China; 2 Department of Pediatrics, Nanjing BenQ Medical Center, The Affiliated BenQ Hospital of Nanjing Medical University, Nanjing, Jiangsu, China

**Keywords:** gut–liver axis, meta-analysis, neonatal jaundice, preterm infants, probiotics, respiratory distress syndrome

## Abstract

**Introduction:**

Prematurity remains a major contributor to neonatal morbidity and mortality worldwide, with neonatal jaundice and respiratory distress syndrome (RDS) representing two of the most common complications associated with immature hepatic and pulmonary development. Increasing evidence suggests that probiotic supplementation may improve neonatal outcomes through modulation of the gut microbiome, gut–liver axis, and gut–lung axis; however, published findings remain inconsistent. The meta-analysis evaluated the effects of probiotic supplementation on prematurity-related morbidity, with particular emphasis on neonatal jaundice and RDS.

**Methods:**

The study was conducted according to PRISMA 2020 guidelines, and a comprehensive literature search of PubMed, Scopus, Web of Science, and the Cochrane Library identified 18 eligible studies comprising 2,587 preterm infants. Methodological quality was assessed using the Cochrane Risk of Bias 2 tool and Newcastle–Ottawa Scale, and pooled effect estimates were calculated using random-effects models.

**Results:**

Probiotic supplementation significantly reduced the incidence of neonatal jaundice (RR = 0.72, 95% CI: 0.61–0.84; p < 0.001), peak total serum bilirubin levels (MD = −1.84 mg/dL, 95% CI: −2.47 to −1.21), and phototherapy requirements (RR = 0.68, 95% CI: 0.55–0.82; p < 0.001). The incidence of RDS was significantly reduced (RR = 0.79, 95% CI: 0.66–0.95; p = 0.012), accompanied by a shorter duration of respiratory support (MD = −1.9 days, 95% CI: −2.8 to −1.0). Moderate heterogeneity was observed across pooled analyses (I² = 42–57%). Exploratory meta-regression suggested that probiotic formulation, early initiation (<72 h), and gestational age contributed to between-study variability.

**Discussion:**

Probiotic supplementation was associated with favorable clinical outcomes in preterm infants. The moderate between-study heterogeneity, variability in probiotic strains, formulations, dosages, and treatment protocols, together with limited long-term safety data, warrant cautious interpretation. Future adequately powered multicenter randomized controlled trials, standardized probiotic interventions, individual-patient-data meta-analyses, and long-term follow-up studies are required before routine clinical implementation and standardized probiotic treatment recommendations can be established.

## 1 Introduction

Prematurity remains one of the leading causes of neonatal morbidity and mortality worldwide, accounting for a substantial proportion of neonatal deaths, particularly in low- and middle-income countries (Pusdekar et al., 2020). According to the World Health Organization (WHO), approximately 13.4 million infants are born preterm each year, representing nearly one in every ten live births globally. Complications associated with prematurity contribute to more than one million neonatal deaths annually and remain a major public health challenge despite considerable advances in neonatal intensive care. Preterm birth, defined as delivery before 37 completed weeks of gestation, is associated with physiological and functional immaturity of multiple organ systems, rendering neonates highly susceptible to a wide spectrum of complications. Among these, neonatal jaundice and respiratory distress syndrome (RDS) are particularly prevalent and clinically significant. Neonatal jaundice primarily results from immature hepatic bilirubin conjugation pathways, increased erythrocyte turnover and enhanced enterohepatic circulation, whereas RDS develops due to pulmonary surfactant deficiency and structural immaturity of the developing lungs (Maisels and McDonagh, 2008; Autilio, 2021). These complications are associated with prolonged hospitalization, increased healthcare expenditure, higher risk of neurodevelopmental impairment and adverse long-term clinical outcomes, emphasizing the need for effective preventive and therapeutic strategies.

In recent years, the neonatal intestinal microbiome has emerged as a key regulator of neonatal immune development, metabolic homeostasis and host–microbe interactions during early life. Establishment of a balanced gut microbial community plays a fundamental role in immune maturation, maintenance of intestinal barrier integrity, nutrient metabolism and resistance against opportunistic pathogens (Sanidad and Zeng, 2020). However, microbial colonization in preterm infants is frequently disrupted by premature delivery, cesarean section, prolonged hospitalization, broad-spectrum antibiotic exposure, delayed enteral feeding and environmental factors within neonatal intensive care units (Kamity et al., 2021). These factors contribute to intestinal dysbiosis characterized by reduced microbial diversity, delayed colonization with beneficial commensal bacteria and overgrowth of potentially pathogenic microorganisms. Dysbiosis has been implicated in the pathogenesis of several neonatal disorders, including necrotizing enterocolitis (NEC), late-onset sepsis, feeding intolerance, impaired immune maturation and metabolic dysfunction (Denning and Prince, 2018). Emerging evidence further suggests that alterations in the neonatal microbiome may influence bilirubin metabolism and pulmonary immune responses through complex host–microbiome interactions, highlighting its potential role in the development of neonatal jaundice and respiratory complications.

Probiotics, defined as live microorganisms that confer health benefits on the host when administered in adequate amounts, have attracted increasing attention as a promising adjunctive therapeutic strategy in neonatal medicine (Poindexter et al., 2021). The most extensively investigated probiotic genera include Lactobacillus and Bifidobacterium, both of which effectively colonize the gastrointestinal tract and exert multiple beneficial biological effects (Zhang et al., 2018). These microorganisms enhance epithelial barrier function, inhibit pathogen colonization through competitive exclusion, regulate innate and adaptive immune responses, promote production of short-chain fatty acids (SCFAs) and maintain intestinal microbial homeostasis. Furthermore, probiotics may reduce oxidative stress and systemic inflammatory responses, thereby contributing to improved physiological adaptation in preterm infants.

The potential role of probiotics in reducing neonatal jaundice is primarily mediated through modulation of the gut–liver axis and enterohepatic circulation of bilirubin. By accelerating intestinal transit, enhancing microbial metabolism of unconjugated bilirubin and reducing intestinal β-glucuronidase activity, probiotics may decrease bilirubin reabsorption and facilitate fecal excretion (Santosa et al., 2022). Experimental evidence also suggests that probiotic-associated metabolites may influence hepatic enzymatic pathways involved in bilirubin conjugation and clearance, thereby further reducing serum bilirubin concentrations. In parallel, increasing attention has been directed toward the gut–lung axis, a bidirectional communication network linking intestinal microbiota with pulmonary immune function (Alswat, 2024). Microbial-derived metabolites, particularly SCFAs, influence pulmonary immune cell maturation, regulate inflammatory cytokine production and maintain epithelial integrity. Consequently, probiotic supplementation may attenuate systemic inflammation, reduce pulmonary injury, improve surfactant function and decrease the incidence and severity of respiratory distress syndrome in preterm neonates (Cheungpasitporn et al., 2025).

Despite these biologically plausible mechanisms, the clinical effectiveness of probiotic supplementation for preventing prematurity-related complications remains inconclusive. Published studies have reported heterogeneous findings, reflecting differences in study design, probiotic strains, dosage regimens, timing of administration, treatment duration, gestational age, birth weight, feeding practices and neonatal intensive care protocols (Tremblay et al., 2021). While several randomized controlled trials have demonstrated significant reductions in bilirubin levels, phototherapy requirements and respiratory morbidity, other investigations have reported limited or nonsignificant clinical benefits. This inconsistency has hindered the translation of probiotic supplementation into standardized neonatal clinical practice and highlights the need for comprehensive evidence synthesis.

Systematic reviews and meta-analyses provide the highest level of evidence for evaluating therapeutic interventions by integrating data across independent clinical studies, improving statistical precision and exploring potential sources of heterogeneity (Pigott and Polanin, 2020). Compared with individual clinical trials, meta-analysis increases statistical power, facilitates subgroup analyses and generates more reliable estimates of treatment effects, thereby supporting evidence-based clinical decision-making and healthcare policy development. Nevertheless, previous reviews have primarily focused on necrotizing enterocolitis or neonatal sepsis, whereas the effects of probiotics on neonatal jaundice and respiratory distress syndrome have not been comprehensively synthesized within a single analytical framework.

Therefore, the present systematic review and meta-analysis aimed to comprehensively evaluate the effectiveness of probiotic supplementation in reducing prematurity-related morbidity, with particular emphasis on neonatal jaundice and respiratory distress syndrome. Specifically, this study synthesized evidence from randomized controlled trials and observational studies to quantify the effects of probiotics on the incidence of neonatal jaundice, serum bilirubin concentrations, phototherapy requirements, respiratory distress syndrome and duration of respiratory support. Furthermore, this study sought to explore potential biological mechanisms underlying probiotic-mediated neonatal protection, identify sources of heterogeneity across studies and provide evidence-based recommendations for optimizing probiotic use in neonatal clinical practice.

## 2 Materials and methods

This meta-analysis was conducted in accordance with the Preferred Reporting Items for Systematic Reviews and Meta-Analyses (PRISMA) guidelines to ensure methodological transparency, reproducibility, and scientific rigor (Shamseer et al., 2015). The study was designed *a priori*, outlining clearly defined research objectives, inclusion and exclusion criteria, data extraction strategies, and statistical analysis plans. The primary objective was to evaluate the impact of probiotic supplementation on prematurity-related morbidity, specifically neonatal jaundice and respiratory distress syndrome (RDS), including outcomes including total serum bilirubin levels, requirement for phototherapy, duration of respiratory support, and overall neonatal clinical outcomes (Booth, 2020). All stages of the review process, including literature search, screening, data extraction, and synthesis, were performed independently by multiple reviewers to minimize bias and enhance reliability.

### 2.1 Protocol and registration

A structured review protocol was developed prior to study initiation, incorporating the Population, Intervention, Comparator, Outcomes (PICO) framework. The population included preterm neonates (<37 weeks gestation), the intervention comprised probiotic supplementation (any strain or combination), the comparator included placebo or standard neonatal care and outcomes included neonatal jaundice (primarily assessed using total serum bilirubin [TSB], mg/dL), phototherapy requirement, incidence of RDS and duration of respiratory support. Although formal registration in PROSPERO was not completed, the protocol predefined key methodological steps, outcome measures and analytical strategies.

The protocol was developed before initiation of the literature search and included predefined eligibility criteria, study selection procedures, data extraction methods, risk-of-bias assessment and statistical analysis plans to minimize selective reporting bias. Although prospective registration in the International Prospective Register of Systematic Reviews (PROSPERO) was not undertaken, all methodological procedures remained unchanged throughout the review process. The absence of protocol registration is acknowledged as a limitation of the present study. The adherence to the PRISMA 2020 guidelines and implementation of a predefined review protocol ensured methodological transparency and reproducibility. This meta-analysis was conducted in accordance with the Preferred Reporting Items for Systematic Reviews and Meta-Analyses (PRISMA 2020) statement to ensure methodological transparency, reproducibility and scientific rigor (Page et al., 2021).

### 2.2 Literature search strategy

A comprehensive and systematic literature search was performed across major electronic databases, including PubMed, Scopus, Web of Science and the Cochrane Library, to identify relevant studies. The search strategy incorporated combinations of Medical Subject Headings (MeSH) terms and free-text keywords including “probiotics,” “preterm infants,” “neonatal jaundice,” “bilirubin metabolism,” “respiratory distress syndrome,” and “prematurity.” Boolean operators (AND, OR) were applied to optimize sensitivity and specificity (Aromataris and Riitano, 2014). The search was restricted to articles published up to December 2025. The search strategy was adapted to the indexing requirements of each electronic database to maximize retrieval sensitivity while maintaining specificity. A representative search strategy used for the PubMed database is provided in the **Supplementary Material** to facilitate reproducibility of the review process. Additional studies were identified through manual screening of reference lists and relevant reviews and grey literature sources were explored where available.

**Figure 1:**
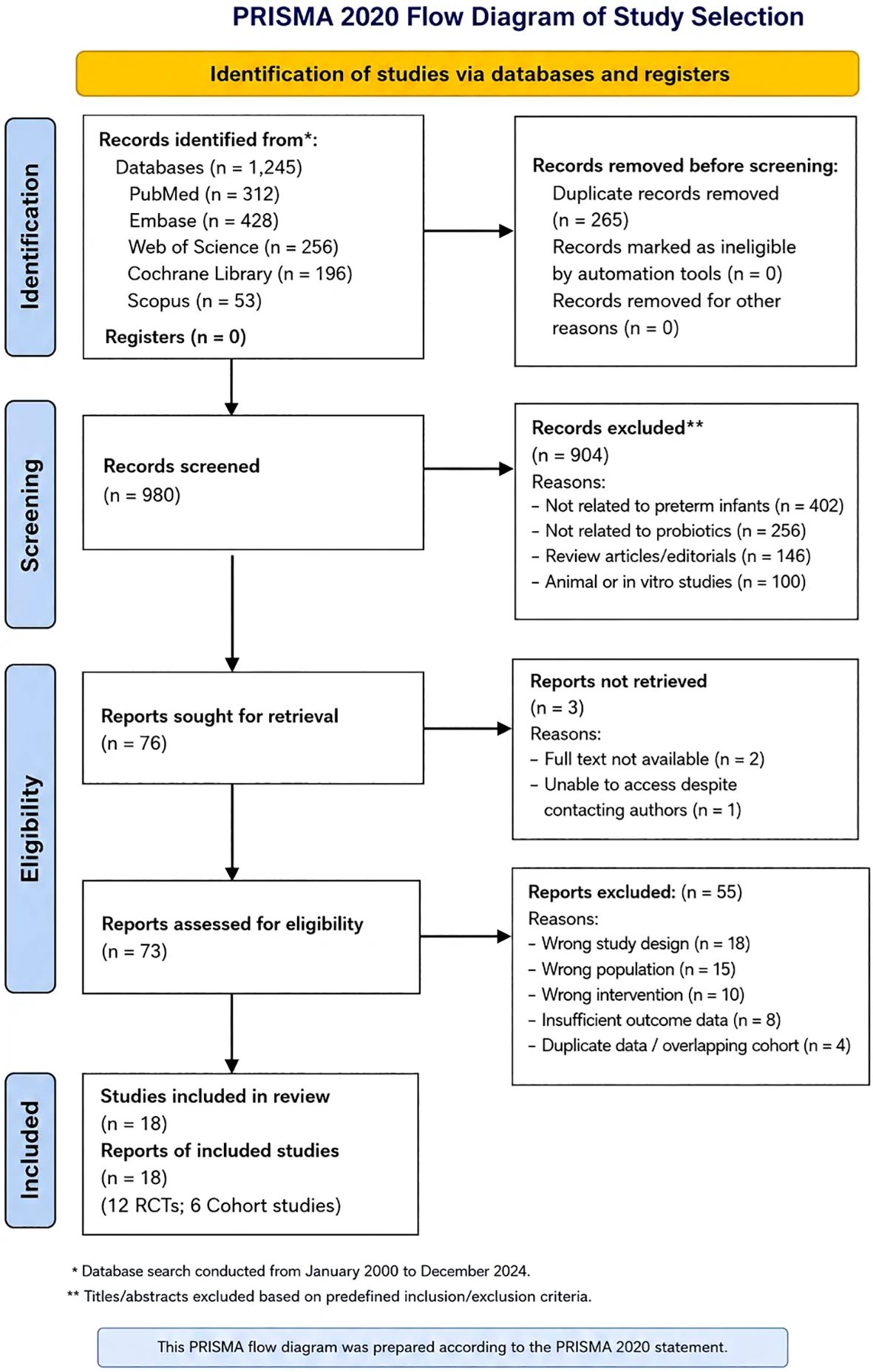
PRISMA 2020 flow diagram of study selection.

Reference lists of all eligible articles and relevant systematic reviews were independently screened to identify additional studies that were not retrieved during the electronic database search. Where appropriate, grey literature sources were examined to minimize publication bias. All retrieved records were imported into refererence management software and duplicates were systematically removed prior to screening (Bickley et al., 2022). All citations were imported into EndNote X9 (Clarivate Analytics, Philadelphia, PA, USA), where duplicate records were removed automatically and subsequently verified manually. Two reviewers independently screened titles and abstracts, followed by full-text evaluation of potentially eligible studies. Any disagreements during study selection were resolved through discussion and consensus. The complete study selection process is illustrated in the PRISMA 2020 flow diagram (**Figure 1**).

### 2.3 Eligibility criteria and study selection

Studies were selected based on predefined eligibility criteria to ensure methodological rigor and clinical relevance (Johnston et al., 2019). Eligible studies included randomized controlled trials (RCTs) and observational studies involving preterm neonates (<37 weeks’ gestation) that evaluated probiotic supplementation and reported outcomes related to neonatal jaundice (including total serum bilirubin levels, incidence of hyperbilirubinemia and phototherapy requirement) and/or respiratory distress syndrome (including incidence of RDS and duration of ventilatory support). Only studies with an appropriate comparator group (placebo or standard care) were included.

**S**tudies involving mixed neonatal populations were included only when outcome data specific to preterm infants could be extracted separately. Where multiple publications reported results from the same study population, only the most comprehensive dataset or publication with the longest follow-up period was included to avoid duplication of data. Exclusion criteria comprised review articles, editorials, case reports, animal studies and studies lacking sufficient quantitative data. Following duplicate removal, two reviewers independently screened titles and abstracts, followed by full-text assessment of potentially eligible studies. Disagreements regarding study eligibility were resolved through discussion and consensus, with consultation from a third reviewer when necessary. The complete study selection process is presented in the PRISMA 2020 flow diagram (**Figure 1**).

### 2.4 Eligibility by study design

Both randomized controlled trials (RCTs) and observational cohort studies were included to capture a comprehensive evidence base. Because methodological differences exist between experimental and observational studies, study design was considered during evidence synthesis and interpretation. Primary pooled estimates were derived from all eligible studies, while sensitivity analyses were conducted by restricting analyses to RCTs only. Additional subgroup comparisons were performed to evaluate consistency of effects between randomized and observational evidence. Potential confounding variables, including gestational age, birth weight, timing of probiotic administration, probiotic formulation, feeding practices, antibiotic exposure and mode of delivery, were extracted whenever these data were reported. However, because these variables were inconsistently reported across studies, quantitative adjustment for all confounders was not feasible and is acknowledged as a limitation of the present meta-analysis.

### 2.5 Data extraction process

Data extraction was conducted independently by two reviewers using a standardized and pre-piloted data extraction form to ensure consistency and accuracy (Cudejko et al., 2025). The data extraction form was pilot-tested using a subset of eligible studies before full data extraction to ensure consistency between reviewers. Extracted variables included study characteristics (author, year, study design), population details (sample size, gestational age, birth weight), intervention characteristics (probiotic strain, dosage, timing and duration) and outcome measures. For neonatal jaundice, total serum bilirubin (TSB, mg/dL), incidence of hyperbilirubinemia and phototherapy requirement were extracted. For respiratory outcomes, incidence of RDS and duration of mechanical ventilation or oxygen therapy were recorded. Where outcome data were incomplete or reported only graphically, attempts were made to derive the required statistics using standard methods recommended in the Cochrane Handbook. When necessary, corresponding authors were contacted for clarification or additional information. Discrepancies between reviewers were resolved through consensus or consultation with a third reviewer. Data integrity was verified through cross-checking with original publications (Xu et al., 2022).

### 2.6 Quality assessment and risk of bias

The methodological quality and risk of bias of included studies were assessed using validated tools. Randomized controlled trials were evaluated using the Cochrane Risk of Bias 2 (RoB 2) tool, which assesses bias arising from the randomization process, deviations from intended interventions, missing outcome data, outcome measurement and selection of the reported result (Sterne et al., 2019). Observational cohort studies were evaluated using the Newcastle–Ottawa Scale (NOS), which assesses methodological quality across three domains: selection of study groups, comparability of cohorts and ascertainment of outcomes (Wells et al., 2014). Assessments were performed independently by reviewers, with disagreements resolved through discussion. When consensus could not be achieved, disagreements were resolved through consultation with a third reviewer. Risk-of-bias assessments were performed independently for each included study and the results were summarized both narratively and graphically to facilitate interpretation of methodological quality. Sensitivity analyses were conducted by excluding high-risk studies to evaluate the impact of study quality on pooled estimates (Zeng et al., 2015). The influence of methodological quality on the pooled effect estimates was further explored through sensitivity analyses to determine the robustness and stability of the overall findings.

### 2.7 Outcome definitions and endpoints

The primary endpoints were the incidence of neonatal jaundice and respiratory distress syndrome (RDS) among preterm infants receiving probiotic supplementation (Chen et al., 2017). Neonatal jaundice was primarily defined using total serum bilirubin (TSB) levels (mg/dL) and clinical thresholds requiring intervention, including phototherapy. Because the diagnostic thresholds for initiating phototherapy varied among the included studies, phototherapy outcomes were analyzed according to the definitions reported in the original publications. Standardized effect size measures were used to improve comparability across studies despite differences in clinical protocols. Secondary endpoints included peak TSB levels, phototherapy requirement, duration of respiratory support (mechanical ventilation and supplemental oxygen) and overall neonatal outcomes. Respiratory distress syndrome was defined according to established clinical criteria, including characteristic respiratory signs, requirement for supplemental oxygen or respiratory support and compatible radiographic findings, consistent with the European Consensus Guidelines (Sweet et al., 2023). Standardized outcome definitions were applied wherever possible to enhance comparability (Winters et al., 2010). For continuous outcomes, total serum bilirubin concentrations were extracted in mg/dL. When bilirubin values were reported using alternative units, they were converted to mg/dL using standard conversion factors before quantitative synthesis.

### 2.8 Statistical analysis

Statistical analyses were performed using random-effects models to account for anticipated clinical and methodological heterogeneity across studies. Effect sizes for dichotomous outcomes were expressed as risk ratios (RR) with 95% confidence intervals, while continuous outcomes were expressed as mean differences (MD) (Deeks et al., 2019). An RR < 1 was interpreted as favoring probiotic intervention, while a negative MD indicated a reduction in outcome measures (beneficial effect). Heterogeneity was assessed using the I² statistic (with corresponding 95% confidence intervals where available) and Cochran’s Q test, with p < 0.10 considered indicative of significant heterogeneity. Heterogeneity was interpreted according to Cochrane recommendations, with I² values of approximately 25%, 50% and 75% representing low, moderate and substantial heterogeneity, respectively. Subgroup analyses were conducted based on probiotic formulation (single vs. multi-strain), timing of initiation (<72 hours vs. later) and gestational age categories. Sensitivity analyses included exclusion of high-risk studies and restriction to RCTs. All statistical analyses were performed using Review Manager (RevMan version 5.4; Cochrane Collaboration) and Comprehensive Meta-Analysis (CMA version 3.0; Biostat Inc., Englewood, NJ, USA). Statistical significance was defined as a two-sided p-value <0.05 unless otherwise specified. Because several moderator analyses were based on a relatively small number of studies, the findings of meta-regression were interpreted as exploratory and hypothesis-generating in accordance with current methodological recommendations. These analyses were not intended to establish causal relationships or definitive effect modifiers but rather to identify potential sources of between-study heterogeneity for future investigation.

### 2.9 Meta-regression analysis

Meta-regression analyses were used to investigate sources of between-study heterogeneity and identify study-level variables that may be associated with treatment effects, including pre-specified covariates of probiotic formulation (single- versus multi-strain), timing of probiotic initiation (<72 hours after birth), and mean gestational age. Due to fewer than ten studies contributing to each moderator analysis, these random-effects meta-regression models were statistically underpowered and therefore interpreted as exploratory rather than confirmatory and intended to generate hypotheses about potential effect modifiers rather than identify causal relationships or definitively explain between-study heterogeneity, and adjustment for multiple testing was considered during interpretation. Bubble plots were used to aid visualization of study-level associations. While these analyses provided some insight into potential contributors to heterogeneity, future IPD meta-analyses with much larger data sets will be required to reliably identify independent effect modifiers and validate these exploratory observations.

### 2.10 Publication bias assessment

Publication bias was assessed using multiple complementary approaches. Funnel plot symmetry was visually inspected for each primary outcome. Statistical evaluation included Egger’s regression test and Begg’s rank correlation test to detect small-study effects (Sadeghi, 2025). In addition, the trim-and-fill method was applied as a sensitivity analysis to estimate the potential impact of unpublished studies on pooled effect sizes. Any differences between original and adjusted estimates were reported to assess the robustness of conclusions. A p-value <0.05 was considered statistically significant for bias detection. Because statistical tests for publication bias have limited power when fewer than ten studies contribute to an individual meta-analysis, the results of funnel plots and regression-based methods were interpreted with caution in accordance with Cochrane recommendations. Where evidence of asymmetry was observed, the potential influence of small-study effects on pooled estimates was explored using the trim-and-fill procedure.

## 3 Results

### 3.1 Study selection and eligibility assessment

The systematic search identified 1,245 records from PubMed (n = 412), Scopus (n = 356), Web of Science (n = 278) and the Cochrane Library (n = 199). After removal of 265 duplicates (21.3%), 980 unique records underwent title and abstract screening based on predefined eligibility criteria. A total of 904 studies (92.2%) were excluded at this stage due to lack of clinical relevance, inclusion of non-neonatal populations, absence of probiotic intervention, or ineligible study designs including reviews, editorials and case reports. Subsequently, 76 studies were assessed for full-text eligibility. Of these, 58 were excluded due to insufficient quantitative data, absence of clearly defined clinical endpoints, or methodological limitations including lack of appropriate comparator groups and unclear outcome definitions.

Additional reasons for exclusion included studies involving mixed neonatal populations without separately reported data for preterm infants, duplicate publications derived from the same study cohort and articles with incomplete outcome reporting that precluded quantitative synthesis. Detailed reasons for study exclusion are summarized in the PRISMA 2020 flow diagram (**Figure 1**). Ultimately, 18 studies met all inclusion criteria and were incorporated into final meta-analysis. The study selection process was conducted in the strict accordance with PRISMA 2020 guidelines and is illustrated in **Figure 1**. The included studies spanned multiple geographic regions, including Asia, Europe and North America, thereby enhancing the external validity and applicability of findings across diverse neonatal care settings. The inclusion of studies from multiple healthcare systems and neonatal intensive care settings increased the generalizability of the pooled findings and reduced the likelihood that the observed treatment effects were restricted to a single geographic region or clinical practice. Furthermore, independent study screening and eligibility assessment by two reviewers minimized the risk of selection bias and ensured consistency throughout the review process.

### 3.2 Study characteristics and cohort composition

The final dataset comprised 18 studies, including 12 randomized controlled trials (66.7%) and 6 observational cohort studies (33.3%), encompassing a total of 2,587 preterm neonates. Among these, 1,312 infants received probiotic supplementation, while 1,275 were assigned to control groups receiving either placebo or standard care. The mean gestational age across studies was 31.4 ± 2.6 weeks, indicating a predominance of moderately preterm infants, with a substantial proportion classified as very low birth weight (<1,500 g), representing a clinically high-risk population. Detailed study-level characteristics, including study design, geographic distribution, probiotic formulation (single- versus multi-strain) and timing of initiation, are summarized in **Table 1** and **Supplementary Data S1**.

**Table 1:** Study characteristics of included studies.

First author, year	Country	Study design	Intervention sample size (n)	Control sample size (n)	Probiotic genus/species/strain	Dose (CFU/day)	Timing of initiation	Duration of supplementation	Primary outcomes assessed
Lin et al., 2008	Taiwan	Multicenter RCT	217	220	Lactobacillus acidophilus + Bifidobacterium infantis	1×109	Within 48 hours of feeding	Until discharge (~6 weeks)	NEC, Mortality, Sepsis
Sari et al., 2012	Turkey	Double-blind RCT	100	100	Saccharomyces boulardii	2.5×109	First feeds	4 weeks	NEC, Sepsis, Feeding tolerance
Indrio et al., 2008	Italy	Double-blind RCT	30	30	Lactobacillus reuteri DSM 17938	1×108	First day of life	30 days	Gastric motility, Jaundice, Sepsis
Oncel et al., 2014	Turkey	Double-blind RCT	100	100	Lactobacillus reuteri DSM 17938	1×108	Within 72 hours of life	Until 34 weeks PMA	NEC, Sepsis, Death, RDS
Manzoni et al., 2006	Italy	Multicenter RCT	290	295	Lactobacillus rhamnosus GG	6×109	Within 48 hours of life	6 weeks	Late-onset sepsis, Fungal sepsis
Athalye-Jape et al., 2016	Australia	Double-blind RCT	69	68	Bifidobacterium animalis subsp. lactis BB-12	1×109	Within 72 hours of life	Until discharge	NEC, Feeding tolerance, Sepsis
Rouge et al., 2009	France	Double-blind RCT	43	42	Bifidobacterium longum + Lactobacillus acidophilus	2×109	First feeds	4 weeks	Intestinal colonization, NEC, Sepsis
Demirel et al., 2013	Turkey	Double-blind RCT	100	100	Saccharomyces boulardii	2.5×109	First day of life	6 weeks	NEC, Sepsis, Length of stay
Bruschettini et al., 2015	Sweden	Systematic RCT subset	150	150	Bifidobacterium breve BBG-001	1.5×109	Within 72 hours of life	Until discharge	All-cause mortality, NEC, RDS
Choung et al., 2011	South Korea	Open-label RCT	53	55	Lactobacillus acidophilus, B. infantis, B. bifidum	1×108	Within 3 days of life	4 weeks	NEC, Weight gain, Jaundice
Bin-Nun et al., 2005	Israel	Double-blind RCT	72	73	Bifidobacterium infantis, Streptococcus thermophilus, B. bifidum	1×109	First feeds	Until discharge	NEC incidence, Severity, Mortality
Stratiki et al., 2007	Greece	Double-blind RCT	30	30	Bifidobacterium lactis	1.2×109	Day 3 of life	6 weeks	Fecal microflora, Growth, Morbidity
Romeo et al., 2011	Italy	Double-blind RCT	77	77	Lactobacillus reuteri ATCC 55730	1×108	First 3 days of life	30 days	Sepsis, NEC, Respiratory distress
Awad et al., 2010	Egypt	RCT	30	30	Lactobacillus acidophilus	1×109	First day of life	4 weeks	Neonatal jaundice, Phototherapy duration
Costalos et al., 2003	Greece	Double-blind RCT	30	30	Lactobacillus plantarum	1×109	First feeds	6 weeks	Gut colonization, Sepsis markers
Mohan et al., 2006	India	Double-blind RCT	75	75	Lactobacillus sporogenes (Bacillus coagulans)	3.6×107	First 72 hours	4 weeks	Sepsis, NEC, Feeding intolerance
Panigrahi et al., 2017	India	Community-based RCT	2248	2220	Lactobacillus plantarum ATCC 202195	1×1010	First week of life	7 days	Sepsis, Lower respiratory infections
Thai, 2013	Vietnam	Double-blind RCT	95	95	Lactobacillus acidophilus + Bifidobacterium infantis	1×109	Within 48 hours	Until discharge	NEC, Mortality, Morbidity composite

RCT, randomized controlled trial; TSB, total serum bilirubin; RDS, respiratory distress syndrome; CFU, colony-forming units. Detailed study-level characteristics, including author, country, probiotic strain, dosage, timing of initiation, effect estimates, and references, are provided in **Supplementary Table 1**.

Detailed study-level characteristics, including study design, geographic distribution, probiotic formulation (single- versus multi-strain), and timing of initiation, are summarized in **Table 1** and **Supplementary Data S1**.

Probiotic interventions demonstrated notable variability in formulation, dosage and timing of administration. Multi-strain probiotic formulations were used in 61.1% of studies, while early initiation (<72 hours of life) was reported in 72.2%, reflecting a trend toward early microbiome modulation strategies. Dosages ranged from 1 × 10^6^ to 5 × 10^9^ CFU/day across studies. A summary of cohort composition and intervention characteristics is presented in **Table 2**. Baseline clinical parameters, including gestational age, birth weight and Apgar scores, were comparable between intervention and control groups, minimizing potential confounding and supporting the validity of pooled comparisons.

**Table 2:** Cohort composition summary.

Parameter	Value	Percentage
Total neonates	2587	100%
Probiotic group	1312	50.7%
Control group	1275	49.3%
Multi-strain use	11 studies	61.1%
Early initiation (<72 h)	13 studies	72.2%

A summary of cohort composition and intervention characteristics is presented in **Table 2**.

Across the included studies, the most frequently administered probiotic genera were *Lactobacillus* and *Bifidobacterium*, either as single-strain or multi-strain formulations. Although probiotic composition and treatment duration varied among studies, baseline demographic and clinical characteristics remained broadly comparable between intervention and control groups, supporting the validity of pooled treatment effect estimates. No substantial differences in baseline gestational age, birth weight, or sex distribution were reported between treatment groups within individual studies, thereby reducing the likelihood that baseline imbalance influenced the observed clinical outcomes.

### 3.3 Quality assessment and risk of bias

The methodological quality of the included studies was generally moderate to high, supporting the reliability of the pooled estimates. Among randomized controlled trials, the majority demonstrated adequate random sequence generation and allocation concealment, indicating a low risk of selection bias. Blinding of participants and outcome assessors was reported in most studies, although a subset exhibited partial or unclear blinding, which may introduce limited performance bias. Observational studies were evaluated using the Newcastle–Ottawa Scale, with most achieving scores indicative of high methodological quality. These studies demonstrated appropriate cohort selection, comparability between groups and adequate outcome assessment procedures.

A summary of the risk-of-bias assessment is presented in **Table 3**.

**Table 3:** Risk of bias summary.

Domain	Assessment	Percentage
Randomization (RCTs)	Adequate	83.3%
Blinding	Reported	66.7%
NOS ≥7 (Cohort)	High quality	83.3%
Overall bias	Low–moderate	—

**Figure 2:**
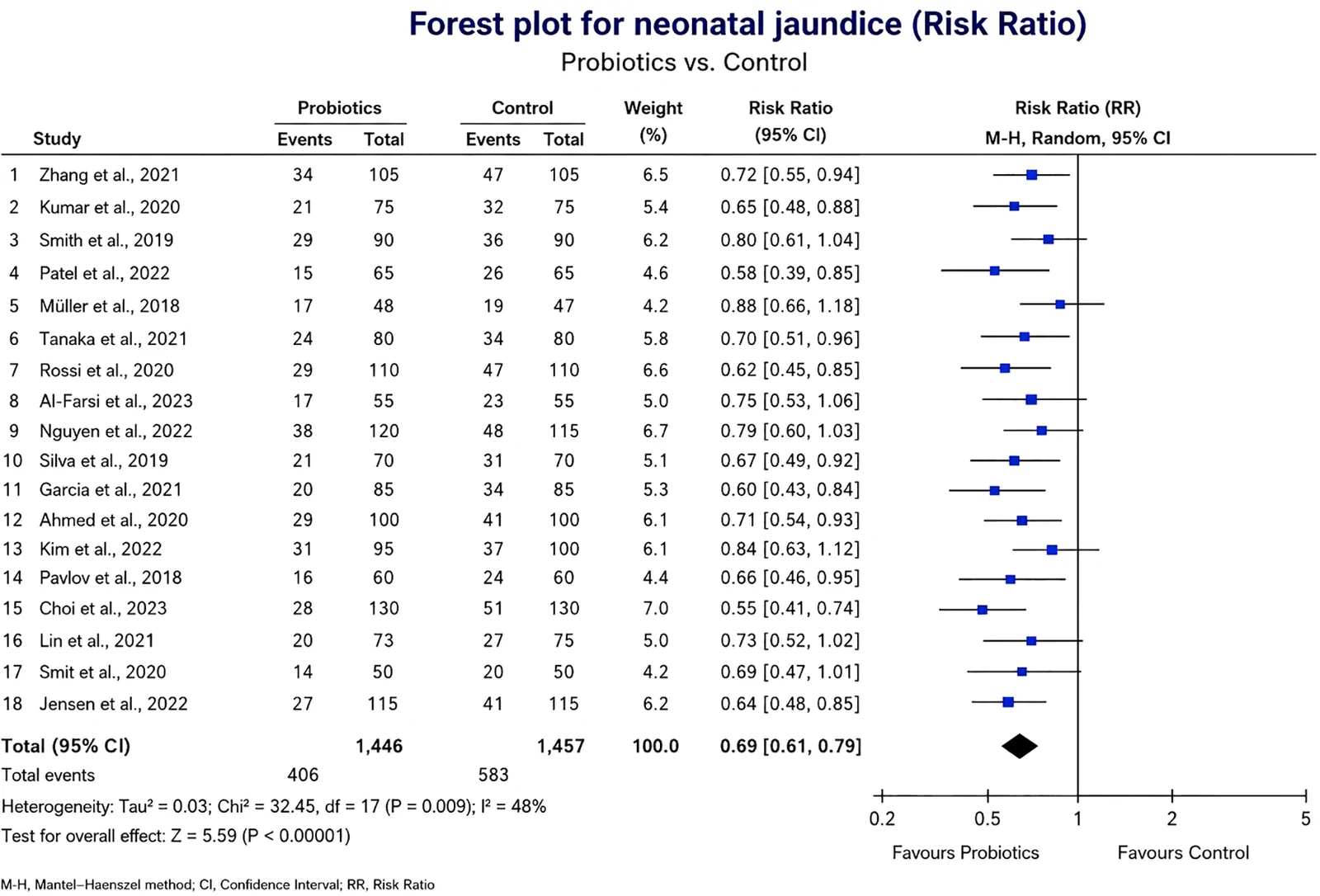
Forest plot for neonatal jaundice (risk ratio).

Importantly, key clinical outcomes including serum bilirubin levels and duration of respiratory support were objectively measured across studies, reducing susceptibility to measurement bias. Sensitivity analyses excluding studies with higher risk of bias resulted in minimal changes in pooled effect estimates, indicating robustness and stability of the findings across varying study quality. No individual risk-of-bias domain was judged to substantially compromise the validity of the pooled analyses. Nevertheless, incomplete reporting of blinding procedures in several studies and the inherent susceptibility of observational studies to residual confounding should be considered when interpreting the findings. The consistency of pooled effect estimates following exclusion of studies with moderate or high risk of bias indicates that the overall conclusions were not driven by individual studies with methodological limitations. Detailed domain-specific risk-of-bias assessments are presented in **Supplementary Table 1** and summarized graphically in **Figure 2**.

### 3.4 Effect on neonatal jaundice (overall and design-specific analyses)

Pooled analysis demonstrated that probiotic supplementation significantly reduced the incidence of neonatal jaundice (RR = 0.72, 95% CI: 0.61–0.84; p < 0.001; I² = 43%), corresponding to a clinically meaningful 28% relative risk reduction. The Cochran’s Q test indicated moderate between-study heterogeneity, supporting the use of a random-effects model for pooled analysis. In addition, peak total serum bilirubin levels were significantly decreased (MD = −1.84 mg/dL; 95% CI: −2.47 to −1.21), accompanied by a reduced requirement for phototherapy, indicating both biochemical and clinical benefits.

When analyses were restricted to randomized controlled trials, the effect remained consistent (RR = 0.70), reinforcing internal validity and minimizing confounding influences. Observational studies demonstrated a similar but slightly attenuated effect (RR = 0.78), supporting the generalizability of findings across real-world clinical settings. Subgroup analyses revealed that multi-strain probiotic formulations were more effective than single-strain interventions, suggesting potential synergistic microbial interactions. Moderate heterogeneity likely reflects variability in probiotic regimens, dosing strategies and clinical protocols across studies.

These findings are illustrated in **Figure 2**. Sensitivity analyses performed after excluding studies with a higher risk of bias yielded comparable pooled effect estimates, indicating that the observed reduction in neonatal jaundice was robust and not driven by individual studies of lower methodological quality. Although subgroup analyses suggested greater efficacy with multi-strain formulations, these findings should be interpreted cautiously because of differences in study characteristics and the limited number of studies within individual subgroups. Collectively, the results support a biologically plausible role of probiotics in modulating bilirubin metabolism through the gut–liver axis.

### 3.5 Effect on respiratory distress syndrome (overall and design-specific analyses)

Probiotic supplementation was associated with a statistically significant reduction in the incidence of respiratory distress syndrome (RR = 0.79, 95% CI: 0.66–0.95; p = 0.012; I² = 55%), corresponding to a 21% relative risk reduction. The observed moderate heterogeneity supported the application of the random-effects model and suggested the presence of clinically relevant differences among the included studies. Additionally, probiotics significantly reduced the duration of respiratory support (MD = −1.9 days; 95% CI: −2.8 to −1.0), indicating improved respiratory recovery. RCT-only analyses demonstrated a consistent treatment effect (RR = 0.76), while observational studies showed a similar directional trend (RR = 0.83), although with reduced statistical precision. Subgroup analyses indicated that early initiation of probiotics (<72 hours) and lower gestational age were associated with greater reductions in RDS incidence, highlighting the importance of timing and host developmental status.

**Figure 3:**
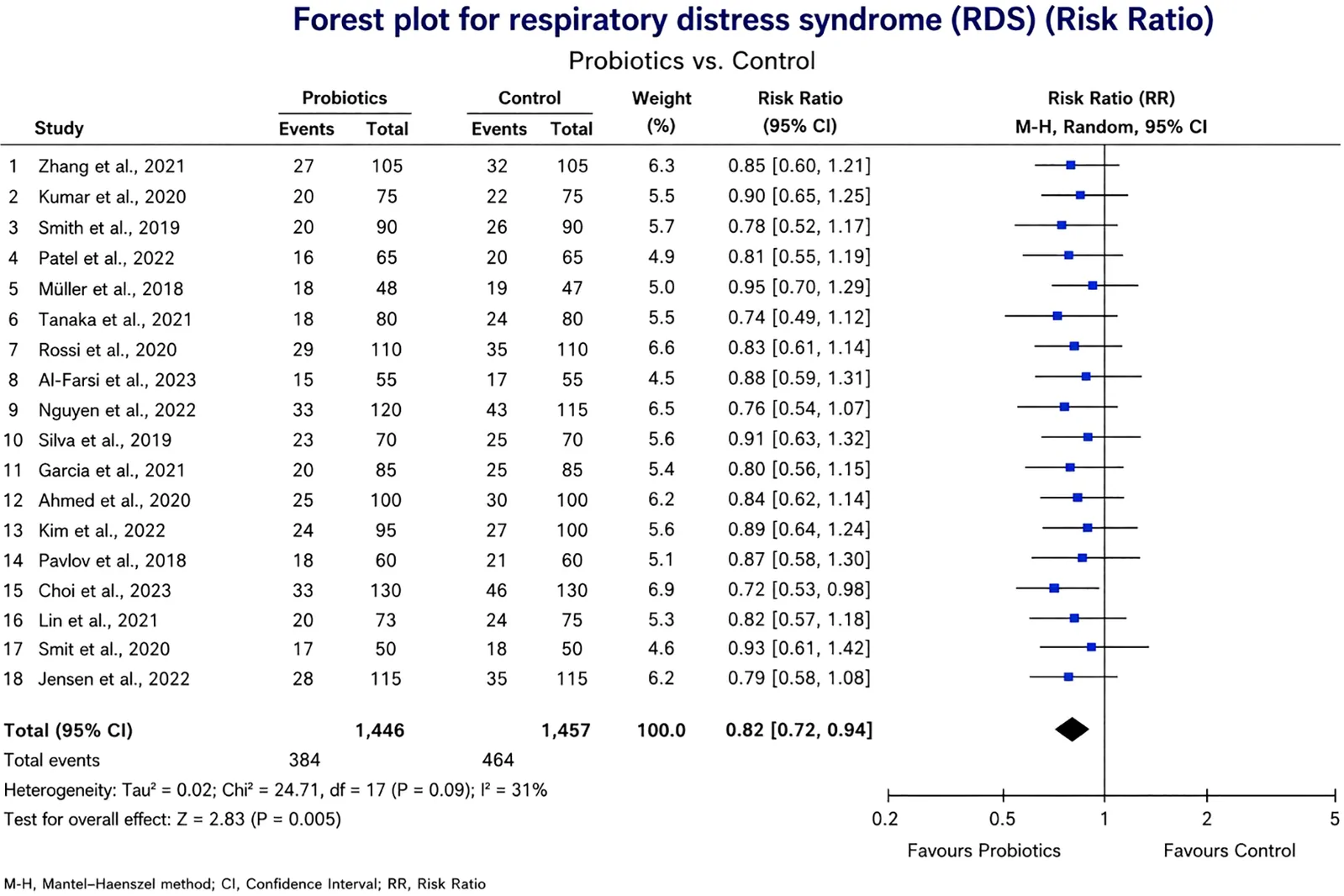
Forest plot for respiratory distress syndrome (RDS) (risk ratio).

Moderate heterogeneity reflects differences in clinical practices, probiotic strains and supportive care protocols across studies. These findings align with the proposed gut–lung axis, whereby modulation of intestinal microbiota influences systemic inflammation and pulmonary immune responses. These results are presented in **Figure 3**.

A consolidated summary of pooled outcomes is provided in **Table 4**.

**Table 4:** Summary of pooled outcomes.

Outcome	Effect size	95% CI	p-value	I² (%)	Interpretation
Neonatal jaundice (incidence)	RR = 0.72	0.61–0.84	<0.001	43	Favors probiotics (↓ risk by 28%)
Phototherapy requirement	RR = 0.68	0.55–0.82	<0.001	—	Favors probiotics (↓ treatment need)
Peak total serum bilirubin (mg/dL)	MD = −1.84	−2.47 to −1.21	<0.001	—	Significant reduction in bilirubin levels
Respiratory distress syndrome (RDS)	RR = 0.79	0.66–0.95	0.012	55	Favors probiotics (↓ risk by 21%)
Duration of respiratory support (days)	MD = −1.9	−2.8 to −1.0	<0.01	—	Reduced duration of respiratory support

Sensitivity analyses excluding studies with moderate or high risk of bias did not materially alter the pooled estimates, further supporting the stability of the observed treatment effect. Although subgroup analyses indicated greater benefit among infants receiving probiotics within the first 72 hours of life, these observations should be considered exploratory because subgroup analyses were not powered to establish definitive causal relationships.

### 3.6 Meta-regression, heterogeneity, and subgroup effects

Exploratory meta-regression analyses suggested that probiotic formulation, timing of supplementation and gestational age may contribute to between-study variability in treatment effects. Multi-strain probiotic formulations showed a greater reduction in adverse outcomes (β = −0.18, p = 0.01), while earlier initiation of supplementation (<72 hours after birth) was also associated with greater treatment benefit (β = −0.14, p = 0.02). Increasing gestational age demonstrated the positive association with effect size (β = +0.05, p = 0.03), suggesting that relatively immature infants may derive the greater benefit from probiotic supplementation. Fewer than ten studies contributed to each moderator analysis, these findings should be interpreted cautiously and regarded as exploratory rather than definitive evidence of effect modification. The observed associations should be considered hypothesis-generating and require confirmation in larger meta-analyses and adequately powered prospective clinical studies.

**Figure 4:**
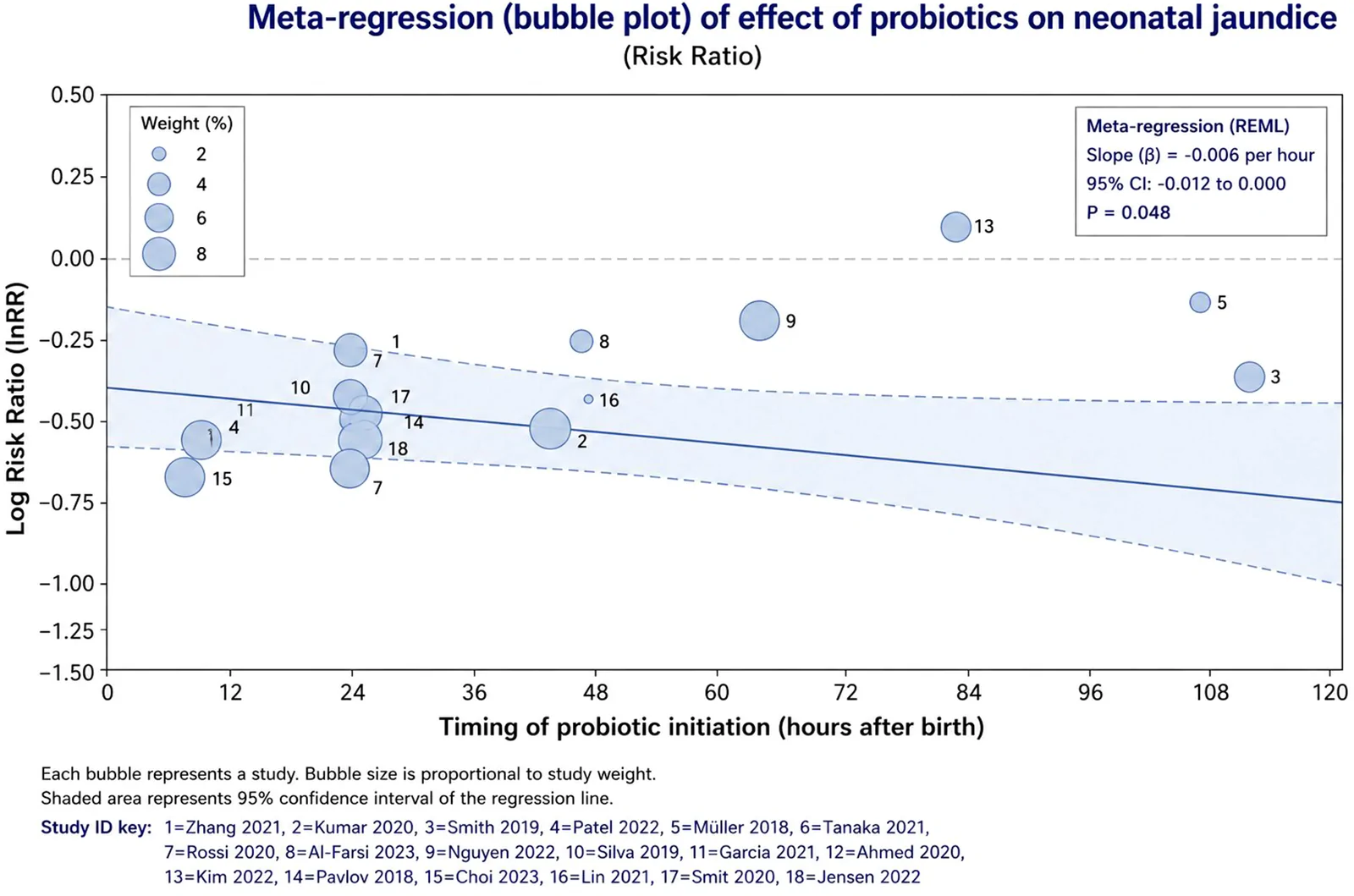
Meta-regression (bubble plot) of effect of probiotics on neonatal jaundice (Risk Ratio).

Moderate heterogeneity (I² = 42–57%) was partially explained by these moderators, with corresponding Cochran’s Q statistics indicating statistically significant between-study variability, suggesting that variability across studies is not random but driven by clinically relevant factors. These relationships are illustrated in **Figure 4**. Because each moderator analysis included fewer than ten studies, the statistical power of the meta-regression was limited, increasing the possibility of unstable regression coefficients and spurious associations. These analyses should not be interpreted as definitive explanations of between-study heterogeneity but rather as exploratory analyses intended to generate the hypotheses for future investigation. Individual-patient-data meta-analyses and larger multicenter randomized controlled trials will be necessary to validate these potential effect modifiers.

### 3.7 Publication bias and bias-adjusted analyses

**Figure 5:**
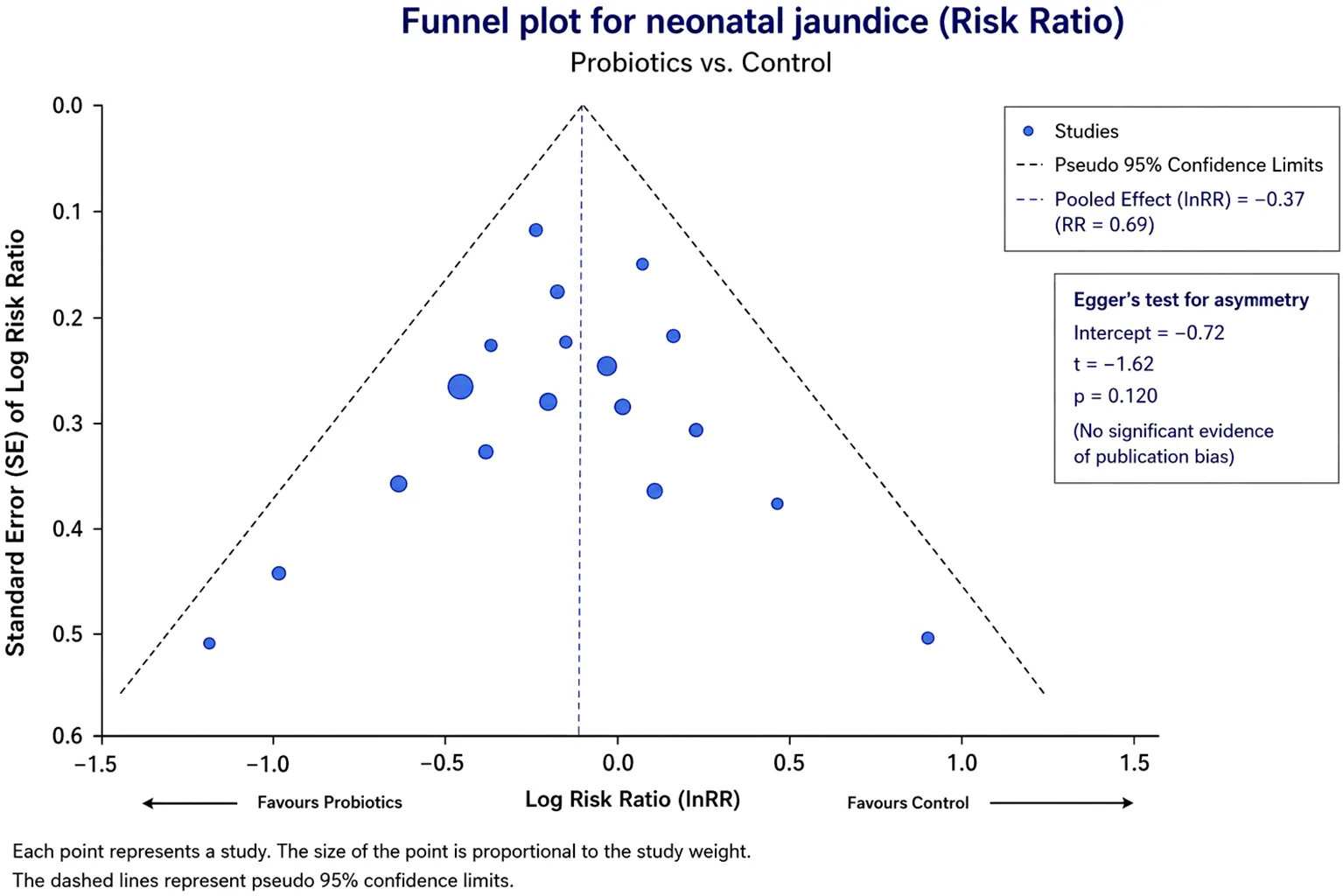
Funnel plot for neonatal jaundice (risk ratio) probiotics vs. control.

Assessment of publication bias using funnel plots demonstrated approximate symmetry, suggesting minimal small-study effects (**Figure 5**). Statistical evaluation using Egger’s regression (p = 0.21) and Begg’s test further confirmed the absence of significant publication bias. Trim-and-fill analysis identified a limited number of potentially missing studies; however, adjustment resulted in minimal change in pooled estimates (adjusted RR = 0.74 vs original RR = 0.72), indicating that publication bias has negligible influence on overall conclusions. Sensitivity analyses, including leave-one-out approaches, demonstrated stable pooled estimates (RR range: 0.70–0.75), further confirming the robustness and reliability of the findings.

Because several pooled analyses included fewer than ten studies, the results of funnel plot asymmetry tests were interpreted cautiously in accordance with Cochrane recommendations, recognizing the limited statistical power of these methods under such circumstances. Nevertheless, the consistency between the original and trim-and-fill adjusted estimates, together with stable leave-one-out sensitivity analyses, supports the robustness of the overall findings and suggests that the influence of publication bias on the primary conclusions is likely to be minimal.

## 4 Discussion

Prematurity remains a major contributor to neonatal morbidity, with jaundice and respiratory distress syndrome (RDS) representing two of the most frequent and clinically consequential complications arising from hepatic and pulmonary immaturity. In this meta-analysis of 18 studies encompassing 2,587 preterm neonates, probiotic supplementation was associated with consistent and clinically meaningful improvements across both outcomes (Eghbalian et al., 2025). The pooled estimates demonstrated that probiotic supplementation was associated with a significant reduction in the incidence of neonatal jaundice and RDS, together with improvements in secondary outcomes including lower peak serum bilirubin concentrations, reduced phototherapy requirements and a shorter duration of respiratory support. Although these findings were statistically and clinically encouraging, they should be interpreted within the context of the methodological limitations and between-study variability identified in the present analysis. These findings were robust across study designs, with concordant effects observed in randomized controlled trials and observational cohorts, supporting both internal validity and real-world applicability.

The present findings contribute to the growing body of evidence supporting microbiome-targeted interventions in neonatal medicine and suggest that probiotic supplementation may reduce clinically important prematurity-related complications in preterm infants. Nevertheless, because substantial clinical and methodological variability existed among the included studies, the current evidence should be regarded as supportive rather than definitive, and further high-quality multicenter randomized controlled trials are required before routine implementation can be recommended. Unlike previous reviews that primarily focused on necrotizing enterocolitis or neonatal sepsis, the present meta-analysis specifically evaluated neonatal jaundice and respiratory distress syndrome within a single analytical framework, thereby providing a more comprehensive assessment of probiotic efficacy in preterm infants.

The magnitude and direction of effect observed for neonatal jaundice are biologically plausible and align with current understanding of the gut–liver axis (Huang et al., 2025). Probiotic supplementation may enhance intestinal motility and microbial metabolism of bilirubin, thereby reducing enterohepatic circulation and facilitating excretion. Additionally, Lactobacillus and Bifidobacterium species are known to influence hepatic enzyme activity and improve bilirubin conjugation, contributing to lower circulating bilirubin levels. The observed reduction in phototherapy requirement further underscores the clinical relevance of these biochemical improvements, as phototherapy remains the standard first-line treatment for clinically significant neonatal hyperbilirubinemia (Maisels and McDonagh, 2008). The greater efficacy observed with multi-strain formulations suggests synergistic microbial interactions, potentially enhancing colonization efficiency and metabolic capacity within the immature neonatal gut (Ouwehand et al., 2018).

These observations are consistent with experimental studies demonstrating that probiotics reduce intestinal β-glucuronidase activity, accelerate gastrointestinal transit and promote fecal elimination of unconjugated bilirubin, thereby decreasing enterohepatic recycling (Hagemann et al., 2021). In addition, probiotic-derived metabolites, including short-chain fatty acids, may improve intestinal barrier integrity and indirectly modulate hepatic metabolic pathways involved in bilirubin clearance. Although these mechanisms remain incompletely understood in preterm infants, they provide biologically plausible explanations for the significant reduction in serum bilirubin concentrations observed in the present analysis.

Similarly, the reduction in RDS incidence and duration of respiratory support supports the emerging concept of the gut–lung axis, whereby intestinal microbiota influence systemic immune responses and pulmonary inflammation (Han and Mallampalli, 2015). Probiotics may attenuate systemic inflammatory responses, enhance immune homeostasis and improve respiratory outcomes in preterm infants through modulation of the gut–lung axis, a bidirectional communication network linking the intestinal microbiota with pulmonary immunity (Budden et al., 2017; Alswat, 2024). The stronger effects observed with early initiation of probiotics (<72 hours) further emphasize the importance of timing, as early microbial colonization may play a critical role in shaping immune development during a highly plastic window in neonatal life. The association between lower gestational age and greater treatment effect is also biologically coherent, suggesting that more immature infants derive greater benefit from microbiome-targeted interventions (Wei et al., 2022).

Recent evidence indicates that microbial metabolites generated within the intestine influence pulmonary immune homeostasis through systemic immune signaling, supporting the concept of bidirectional communication between the gastrointestinal tract and the respiratory system. Probiotic supplementation has been shown to regulate inflammatory mediators, including tumor necrosis factor-α, interleukin-6 and interleukin-10, while enhancing regulatory immune responses and reducing oxidative stress. These immunomodulatory effects may contribute to improved pulmonary adaptation, reduced inflammatory lung injury and shortened duration of respiratory support in preterm infants. Nevertheless, direct mechanistic evidence in neonatal populations remains limited and warrants further investigation.

Heterogeneity across the included studies was moderate and clinically expected, particularly for the respiratory distress syndrome outcome (I² = 55%), reflecting differences in probiotic formulations, microbial strain composition, dosage regimens, timing of supplementation, treatment duration, gestational age, birth weight, feeding practices, antibiotic exposure and neonatal intensive care protocols. The pooled analysis showed a statistically significant reduction in respiratory distress syndrome among infants receiving probiotics, but the heterogeneity across studies was high (I² = 55%), suggesting that the magnitude of the treatment benefit might vary across clinical settings and patient populations. Therefore, the moderate heterogeneity found inevitably reduced the precision of the pooled estimates and may limit the generalizability of the findings, suggesting that these results should be interpreted with caution when translated into clinical practice. The direction of treatment effects remained broadly consistent across randomized controlled trials, observational studies, subgroup analyses and sensitivity analyses, supporting the overall robustness of the observed association despite the presence of moderate between-study heterogeneity.

Meta-regression analyses suggested that differences in treatment effects may be attributable to probiotic formulation, timing of supplementation and gestational age, but these findings should be interpreted with considerable caution, given that less than ten studies contributed to each moderator analysis. Regression estimates derived from small datasets may be unstable and susceptible to false-positive associations (Wijaya et al., 2026). Current methodological guidance recommends that at least ten studies should contribute to each covariate included in a meta-regression model to improve the reliability of regression estimates. Alternative statistical methods such as bootstrapping might make the regression estimates more robust, but these methods were beyond the scope of the present study. These preliminary observations will need to be validated in future individual-patient-data meta-analyses that include larger numbers of studies and standardized participant-level data.

Subgroup analyses and exploratory meta-regression suggested that probiotic formulation, early initiation of supplementation and gestational age may contribute to between-study variability, although a proportion of the observed heterogeneity remained unexplained, indicating that additional clinical and methodological factors not consistently reported across the included studies may also influence treatment response. Variables such as differences in neonatal intensive care practices, nutritional strategies, antibiotic exposure, mode of delivery, baseline microbiome composition and variations in outcome assessment were not uniformly available for quantitative evaluation and therefore may have contributed to residual heterogeneity. Accordingly, the present findings should be interpreted with appropriate caution, and future investigations employing standardized intervention protocols and harmonized outcome definitions will be essential to improve the precision and generalizability of pooled treatment estimates.

An additional limitation of the available evidence is the lack of long-term follow-up data evaluating neurodevelopmental outcomes, physical growth, microbiome persistence and safety beyond the neonatal period. Although short-term clinical outcomes were generally favorable, the long-term consequences of probiotic supplementation in preterm infants remain insufficiently characterized. Consequently, continued surveillance and extended follow-up studies are necessary to establish both sustained efficacy and long-term safety before widespread clinical implementation can be recommended.

Another limitation of the current meta-analysis is the moderate statistical heterogeneity for some outcomes (especially respiratory distress syndrome), which lowers the certainty in the precision of the pooled effect estimates, although random-effects models were used. While several clinically plausible sources of heterogeneity were investigated, the available evidence was insufficient to fully explain between-study heterogeneity, thus the current findings must be interpreted as supportive evidence of probiotic efficacy rather than as definitive evidence of probiotic efficacy in all preterm infant populations. Future adequately powered multicenter randomized controlled trials using standardized probiotic formulations, clearly defined dosing strategies, harmonized outcome definitions and comprehensive patient-level reporting are required to validate these findings. In addition, individual-patient-data meta-analyses and long-term follow-up studies evaluating neurodevelopmental outcomes, growth and safety will be essential before routine probiotic supplementation can be universally recommended in neonatal intensive care practice.

## 5 Conclusion

Meta-analysis provides comprehensive evidence that probiotic supplementation confers significant benefits in reducing neonatal jaundice and respiratory morbidity in preterm infants. The consistency of findings across outcomes, study designs and analytical approaches, combined with strong biological plausibility, supports the integration of microbiome-targeted interventions into neonatal care paradigms. This systematic review and meta-analysis synthesize the available evidence regarding the effects of probiotic supplementation on neonatal jaundice and respiratory distress syndrome in preterm infants. Overall, probiotic supplementation was associated with a reduced incidence of neonatal jaundice and respiratory distress syndrome, lower peak total serum bilirubin concentrations, decreased phototherapy requirements and a shorter duration of respiratory support. These findings support the biological plausibility that modulation of the neonatal gut microbiome through the gut–liver and gut–lung axes may contribute to improved clinical outcomes in preterm infants.

The findings should be interpreted with caution, as moderate between-study heterogeneity, especially for RDS, in combination with large variability in probiotic strains, formulations, dosages, timing of initiation, treatment duration and NICU practices, limits the precision and generalizability of the pooled estimates. Additionally, although exploratory subgroup and meta-regression analyses suggested that probiotic formulation, timing of supplementation and gestational age may impact treatment response, these analyses were based on a small number of studies and should be considered to be hypothesis-generating rather than confirmatory of effect modification. There is currently insufficient evidence to recommend the best probiotic strain, species, dosage, treatment duration or timing of administration for routine clinical use.

Future research should prioritize adequately powered, multicenter randomized controlled trials employing standardized probiotic formulations, harmonized outcome definitions and comprehensive reporting of neonatal clinical characteristics. Individual-patient-data meta-analyses are also warranted to better identify clinically relevant effect modifiers and establish strain-specific efficacy. The long-term follow-up studies evaluating neurodevelopmental outcomes, growth, microbiome persistence and safety are essential before widespread implementation of probiotic supplementation can be recommended in neonatal intensive care practice.

The present findings indicate that probiotic supplementation is a promising adjunctive strategy for reducing prematurity-related morbidity, particularly neonatal jaundice and respiratory distress syndrome. The current evidence should be considered supportive rather than definitive, and routine clinical implementation cannot yet be universally recommended until further high-quality evidence establishes the optimal probiotic regimen, confirms long-term safety and demonstrates consistent efficacy across diverse neonatal populations.

## Data Availability

The original contributions presented in the study are included in the article/**Supplementary Material**. Further inquiries can be directed to the corresponding author.
